# Supplementary material for: Synaptic and transcriptionally downregulated genes are associated with cortical thickness differences in autism
Source: Mol Psychiatry. 2018 Feb 26;24(7):1053–64. doi: 10.1038/s41380-018-0023-7 (PMC6755982; doi:10.1038/s41380-018-0023-7)
Supplement: Supplementary file 1 — Supplementary material [file 41380_2018_23_MOESM1_ESM.docx]

Synaptic and transcriptionally downregulated genes are associated with cortical thickness differences in children with autism

Romero-Garcia, R., Warrier, V., Bullmore, E.T., Baron-Cohen, S. & Bethlehem R.A.I.

Supplementary Note

1. Neuroimaging Data 2

Discovery dataset 2

Overview 2

Quality control and matching 2

Validation Dataset 2

2. Gene Expression Data 3

3. PLSR analysis 4

Overview 4

4. PLSR Analysis: Autism Data 6

Discovery dataset 6

Table S1: 35 component cross-validation 6

Figure S1: Variance in gene expression explained by components in the Discovery dataset 7

Table S2: Kegg 2016 Pathway analysis for PLSR1 7

Validation datasets 7

Figure S2: Variance in gene expression explained by the first six components in the two Validation datasets 8

Figure S3: Correlations between gene loadings in all three datasets 8

Table S3A: Top 10 pathways (Validation 1) 9

Table S3B: Top 10 pathways (Validation 2) 9

Figure S4: Correlations between ΔCT, gene scores and gene loadings between the Discovery and the males-only Validation 2 10

5. Von Economo profiling 10

Figure S5: Von Economo Expression Z-Scores of the two validation datasets 11

6. PLSR Analysis: ADHD data 11

Table S5: PLS 35 component model for the ADHD-controls CT difference 12

7. Gene modules and enrichment analyses 12

Transcriptional dataset and adult gene co-expression modules 12

Validation Transcriptional dataset 13

Developmental gene co-expression modules 14

Rare de novo genetic variants 14

Common genetic variants 14

Regression analyses 15

Table S6: Results of the gene enrichment analyses 15

8. Third validation dataset 17

Figure S6: third validation dataset 17

References 18

## 1. Neuroimaging Data

### Discovery dataset

#### Overview

A description of the dataset is provided in detail elsewhere (Bethlehem et al., 2017). Briefly, structural T1-weighted MPRAGE images were collected from two publicly available datasets: ABIDE (<http://fcon_1000.projects.nitrc.org/indi/abide/)> and ADHD-200 (<http://fcon_1000.projects.nitrc.org/indi/adhd200/)>. From these datasets, we selected a subset of 3 diagnostic groups (autism, ADHD and neurotypical individuals) of males between the ages of 8 and 12 years old. The initial sample consisted of 348 eligible individuals (see below for details on sample matching and quality control). The structural T1-MPRAGE data were pre-processed using Freesurfer *v5.3* to estimate regional cortical thickness. The cortical thickness maps were automatically parcellated into 308 equally sized cortical regions of 500 mm^2^ that were constrained by the anatomical boundaries defined in the Desikan-Killiany atlas (Desikan et al., 2006; Romero-garcia et al., 2012). Individual parcellation templates were created by warping this standard template containing 308 cortical regions to each individual MPRAGE image in native space. A key advantage of warping of the segmentation map to the native space relates to the attenuation of possible distortions from warping images to a standard space that is normally needed for group comparisons. Lastly, average cortical thickness was extracted for each of the 308 cortical regions in each individual participant.

#### Quality control and matching

Details of the quality control and matching are described in detail elsewhere (Bethlehem et al., 2017). In short, scans were visually checked by two independent researchers. When both researchers independently agreed that the scan quality was good, the subjects were included in the final sample. Variance in cortical thickness across all subjects was also analysed and subjects with variance in global cortical thickness that was more than 3 standard deviations from the sample mean were removed from subsequent analysis. After this last step, there were a few sites that only contained 2 or fewer subjects (MaxMun, Olin, Pitt, Stanford and Trinity) and, to minimize the effect of regressing out site, these sites were removed from subsequent analyses. Secondly, two sites had more than 10 subjects selectively from only one group (Washington University and Peking), as regressing out these sites would effectively also remove potential group effects these sites were removed from further analyses. This left a total sample size of 218 subjects: ADHD *(n=69, age = 9.99 ±1.17, IQ = 107.95 ±14.18)*, autism *(n=62 age=10.07 ±1.11, IQ = 108.86 ±16.94)* and controls *(n=87, age = 10.04 ±1.13, IQ = 110.89 ±10.39)*.

### Validation Dataset

In order to validate our findings, we used two independent datasets of children with and without autism in a similar age range from the second release of ABIDE (<http://fcon_1000.projects.nitrc.org/indi/abide/abide_II.html>). Specifically, we utilized the data collected at Georgetown University (Validation 1) and Kennedy Krieger Institute (Validation 2) that were not included in the first release of ABIDE and consists of a young cohort of children with and without autism. Structural T1-weighted MPRAGE images were pre-processed with the same pipeline as described above. Subjects that had an overall variance in CT that was more than 3 standard deviations from the group mean were removed from further analysis. The final sample for validation consisted of 102 subjects for Validation 1 [autism (*n=48, age=10.97±1.53*) and controls (*n=54, age=10.43±1.71*)] and 21o subjects for Validation 2 [autism (*n=56, age=10.32±1.51*) and controls (*n=154, age=10.34±1.20*)].

## 2. Gene Expression Data

A gene expression dataset of the adult human brain created by the Allen Institute for Brain Science (AIBS; http://human.brain-map.org) (Hawrylycz et al., 2015, 2012) was used to determine the expression profile of each cortical region. This dataset includes samples from post-mortem brain of six donors (3 Caucasian, 2 African-American, 1 Hispanic) aged 24-57 years. The limited sample size (n=6) and the large variability in age, gender and ethnicity may have a deep impact in the regional transcriptional pattern. In order to address the potential inter-individual differences of gene expression we tested the effect of donor selection using a leave-one-donor-out approach. Thus, gene expression values were recalculated six times leaving one different donor out. We found that the six resulting gene expression profiles were highly similar, showing a relative difference (defined as [uncorrected – corrected]/corrected) of 2.53%, 1.98%, 0.31%, 1.09% and 1.20%. In the same line, gene expression of the complete dataset (six donors) and the values after the removal of each donor were strongly associated, showing an r-value of 0.81, 0.79, 0.93, 0.90, 0.87 and 0.90, when donors 1 to 6 were removed from the data. The consistency of gene expression across donors confirms that results reported in present study are not driven by a single donor.

Beside the effect of each individual donor, batches used across different assemblies of the AIBS can account for a large amount of variance in microarray probes. Here, we tested the robustness of our gene expression values to the effect of artefactual correlation induced by batch and donor effects by using Combining Batches of Gene Expression Microarray Data (ComBat; Johnson et al., 2007). Combat has shown higher overall performance than other commonly used methods (Chen et al., 2011). We found that corrected and uncorrected gene expression values were highly similar, showing relative differences of 3.58%, 3.12%, 2.76%, 3.03%, 3.99% and 2.80%, for each of the six donors, and an average r-value of 0.996. The low impact of batch correction in the resulting values reveals that the expression levels are not driven by non-uniform batch sampling.

## 3. PLSR analysis

### Overview

*The following describes a simplified overview of PLSR and the difference between the SIMPLS and the NIPALS algorithm.*

Partial least squares regression or PLSR is a data reduction technique closely related to principal component analysis (PCA) and ordinary least squares (OLS) regression. Here we use the SIMPLS algorithm (de Jong, 1993), where the independent variable matrix (X) and the dependent variable (Y) is centred giving rise to X_0_ and Y_0_ respectively. The first component is then weighted by w_1_ and q_1_ to calculate factor scores (or component scores) T_1_ and U_1_.

This T1 is the weighted sum of the centred independent variable:

*T_1_ = X_0_w_1_ + E_1_* (eqn 1)

And U1 is the weighted sum of the centred dependent variable:

U_1_ = Y_0_q_1_ + E_2_ (eqn 2)

The weights and the factors scores are calculated to ensure the maximum covariance between T_1_ and U_1_, which is a departure from regular PCA where the scores and loadings are calculated to explain the maximum variance in X_0_.

*So U_1_ ~ T_1_* (eqn 3)

Or,

*U_1_ = B_0_ + B_1_T_1_ + E_4_* (eqn 4)

Or,

*U_1_ = B_0_ + B_1_(X_0_w_1_) + E_5_* (eqn 5)

In the SIMPLS algorithm provides an alternative where the matrices are not deflated by the weights when calculating the new components, and, as a result, it is easier to interpret the components based on the original centred matrices.

As the components are calculated to explain the maximum covariance between the dependent and independent variable, the first component need not explain the maximum variance in the dependent variable. However, as the number of components calculated increases, they progressively tend to explain lesser variance in the dependent variable.

*Here we present the rationale for choosing genes with both positive and negative weights:*

From equations 2 and 5 above, we know that:

*Y_0_q_1_ =* *B_0_ + B_1_(X_0_w_1_) + E_5_* (eqn 6)

This can be rewritten as:

*Y_0_q_1_ ~ B_1_(X_0_w_1_)* (eqn 7)

And if both B_1_ and q_1_ are positive which is the case in our analyses, then,

*Y_0_ ~ X_0_w_1_*  (eqn 8)

In our dataset:

Y_0_ is a px1 vector of ΔCT with positive and negative values.

q_1_ is a 1x1 vector of weight for the first PLSR component.

B1 is the regression coefficient.

X_0_ is a pxn matrix of gene expression, where p is the number of cortical regions, and n is the number of genes for which gene expression is calculated. This has been scaled and normalized to have positive and negative values. Positive values indicate that the gene is overexpressed compared to the mean gene expression, and negative values indicate that the gene is underexpressed compared to the mean gene expression.

w1 is a nx1 vector of weights for the first PLSR component.

Y_0_ can be both positive or negative (ΔCT is both positive or negative, as some regions are thicker in individuals with autism compared to controls and vice versa). Similarly, both X_0_ and w_1_ are positive or negative.

This gives us the following possibilities:

1. For a negative value in Y_0_, either the equivalent X_0_ value or the equivalent w_1_ value must be negative.
2. For a positive value in Y_0_, both the equivalent values in X_0_ and w_1_ must be either positive or negative.

In other words, if the weight of the gene is positive, having a higher than average gene expression (positive X_0_) contributes to positive ΔCT (i.e. greater CT in autism compared to controls), whereas having a lower than average gene expression (negative X_0_) contributes to negative ΔCT. Similarly, if the weight of the gene is negative, having a higher than average gene expression contributes to negative ΔCT, whereas having a lower than average gene expression contributes to positive ΔCT. So, the sign of the weights alone cannot tell us if the gene contributes to thicker or thinner cortex in autism compared to controls. It is the combination of both the weights and the gene expression level that can be informative. However, as gene expression and ΔCT varies considerably across the regions tested, we used genes with both positive and negative weights, that were significant FDR correction in our enrichment analyses.

## 4. PLSR Analysis: Autism Data

### Discovery dataset

Details of the PLSR analysis are described in the main manuscript, Supplementary Table S1 below lists the descriptive cross-validation statistics of the full 35-component model and Supplementary Figure S1 shows the amount of explained variance for each component included in the final analysis. Only the first component showed a significant effect (p = 0.009). We also conducted KEGG pathway enrichment analysis, details of which are provided in Supplementary Table S2.

##### Table S1: 35 component cross-validation

| **COMP** | **PRESS** | **RSS** | **Q2** | **Q2cum** | **RMSE** |
| --- | --- | --- | --- | --- | --- |
| 1 | 2.97E+02 | 3.07E+02 | 0.0336 | 0.0336 | 0.9984 |
| 2 | 2.65E+02 | 2.69E+02 | 0.0165 | 0.0496 | 0.9350 |
| 3 | 2.08E+02 | 2.32E+02 | 0.1003 | 0.1449 | 0.8673 |
| 4 | 1.65E+02 | 1.79E+02 | 0.0813 | 0.2145 | 0.7633 |
| 5 | 1.35E+02 | 1.45E+02 | 0.0736 | 0.2722 | 0.6867 |
| 6 | 1.17E+02 | 1.25E+02 | 0.0629 | 0.3180 | 0.6376 |
| 7 | 8.84E+01 | 9.84E+01 | 0.1013 | 0.3871 | 0.5652 |
| 8 | 6.88E+01 | 6.90E+01 | 0.0031 | 0.3890 | 0.4732 |
| 9 | 4.66E+01 | 5.20E+01 | 0.1033 | 0.4521 | 0.4109 |
| 10 | 4.15E+01 | 4.28E+01 | 0.0303 | 0.4687 | 0.3726 |
| 11 | 3.09E+01 | 3.24E+01 | 0.0474 | 0.4939 | 0.3243 |
| 12 | 2.51E+01 | 2.63E+01 | 0.0457 | 0.5170 | 0.2921 |
| 13 | 2.09E+01 | 2.16E+01 | 0.0300 | 0.5315 | 0.2647 |
| 14 | 1.58E+01 | 1.56E+01 | -0.0157 | 0.5241 | 0.2248 |
| 15 | 1.11E+01 | 1.08E+01 | -0.0268 | 0.5114 | 0.1876 |
| 16 | 7.76E+00 | 7.68E+00 | -0.0107 | 0.5062 | 0.1579 |
| 17 | 6.18E+00 | 5.97E+00 | -0.0356 | 0.4886 | 0.1392 |
| 18 | 4.44E+00 | 4.43E+00 | -0.0027 | 0.4872 | 0.1199 |
| 19 | 3.41E+00 | 3.32E+00 | -0.0252 | 0.4743 | 0.1039 |
| 20 | 2.71E+00 | 2.67E+00 | -0.0158 | 0.4659 | 0.0931 |
| 21 | 2.11E+00 | 1.83E+00 | -0.1505 | 0.3856 | 0.0772 |
| 22 | 1.53E+00 | 1.31E+00 | -0.1671 | 0.2829 | 0.0653 |
| 23 | 9.77E-01 | 8.81E-01 | -0.1093 | 0.2045 | 0.0535 |
| 24 | 7.11E-01 | 6.07E-01 | -0.1703 | 0.0690 | 0.0444 |
| 25 | 4.14E-01 | 3.71E-01 | -0.1157 | -0.0386 | 0.0347 |
| 26 | 2.77E-01 | 2.71E-01 | -0.0244 | -0.0640 | 0.0296 |
| 27 | 1.99E-01 | 1.79E-01 | -0.1095 | -0.1805 | 0.0241 |
| 28 | 1.31E-01 | 1.13E-01 | -0.1599 | -0.3692 | 0.0191 |
| 29 | 8.53E-02 | 6.65E-02 | -0.2834 | -0.7572 | 0.0147 |
| 30 | 5.07E-02 | 4.42E-02 | -0.1459 | -1.0136 | 0.0120 |
| 31 | 4.12E-02 | 3.10E-02 | -0.3285 | -1.6751 | 0.0100 |
| 32 | 2.83E-02 | 2.04E-02 | -0.3876 | -2.7119 | 0.0081 |
| 33 | 1.81E-02 | 1.41E-02 | -0.2831 | -3.7627 | 0.0068 |
| 34 | 1.24E-02 | 9.81E-03 | -0.2688 | -5.0428 | 0.0056 |
| 35 | 7.48E-03 | 5.97E-03 | -0.2536 | -6.5753 | 0.0044 |

*For each component the Predictive Error Sum of Squares (PRESS), Residual Sum of Squares (RSS), cross validated PRESS (Q2), the cumulative Q2 and the Root Mean Square of the Error (RMSE) are provided.*

##### Figure S1: Variance in gene expression explained by components in the Discovery dataset


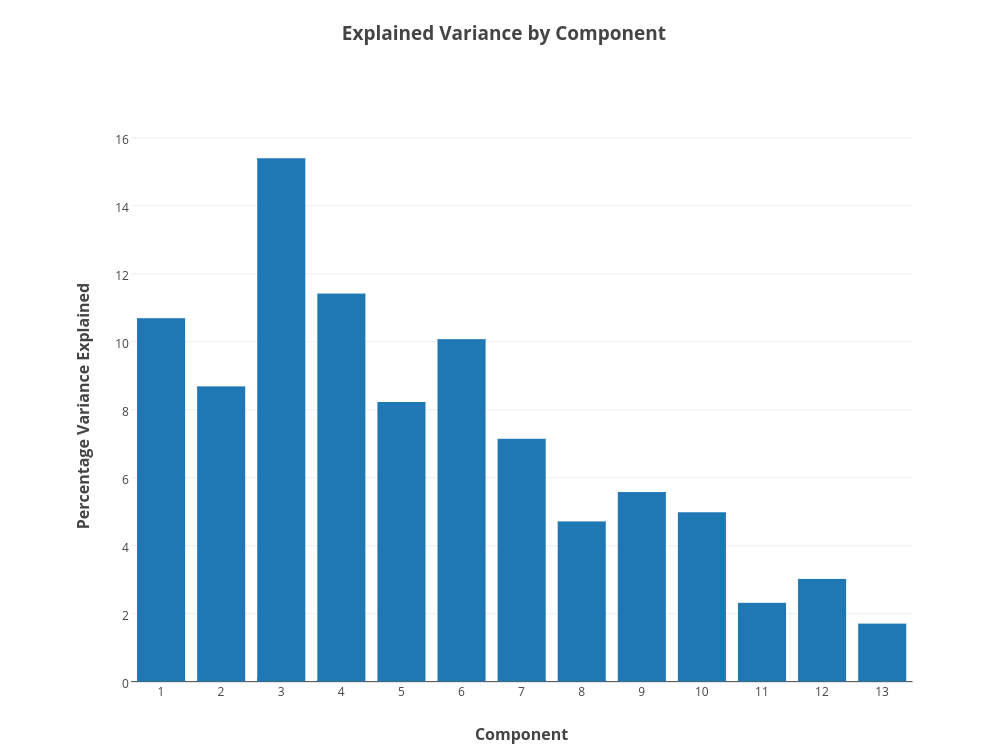


*Variance explained for all 13 components included in the final model. Only components 1, 3, 4 and 6 explained more than 10% of the total variance individually, and were thus selected for further analyses. Of these 4 only component 1 explained a significant proportion of the variance in ΔCT.*

##### Table S2: Kegg 2016 Pathway analysis for PLSR1

| Term | Overlap | P-value | Adjusted P-value | Z-score |
| --- | --- | --- | --- | --- |
| Retrograde endocannabinoid signaling | 29/101 | 8.64092E-07 | 0.000121837 | -1.912944439 |
| GABAergic synapse | 27/88 | 4.79644E-07 | 0.000121837 | -1.856183708 |
| Adrenergic signaling in cardiomyocytes | 36/148 | 3.5192E-06 | 0.000330805 | -1.809256835 |
| Morphine addiction | 25/91 | 1.13401E-05 | 0.000799478 | -1.774306875 |
| Dopaminergic synapse | 29/129 | 0.00013772 | 0.006472825 | -1.761079175 |
| HIF-1 signaling pathway | 25/103 | 0.000107567 | 0.006066762 | -1.73577233 |
| Nicotine addiction | 13/40 | 0.000234762 | 0.009457564 | -1.56949222 |
| Serotonergic synapse | 25/112 | 0.000432338 | 0.015239928 | -1.69105338 |
| Circadian entrainment | 22/95 | 0.000547828 | 0.01716529 | -1.71333288 |
| Oxytocin signaling pathway | 31/158 | 0.001019701 | 0.028755556 | -1.73576073 |
| Renin secretion | 16/64 | 0.001271626 | 0.032599873 | -1.522988352 |
| Pathways in cancer | 62/397 | 0.003043863 | 0.053648094 | -1.709378099 |

### Validation datasets

For the two validation datasets, we conducted the same analysis. For Validation1, the cross-validation analysis identified that 15 components provide the best model fit. For validation 2, the cross-validation analysis identified that 16 components provide the best model fit. Only the first component explained a significant amount of variance (P< 10^-14^) in both the validation datasets, and was thus analyzed further. As was the case in the discovery dataset the first component in both the datasets was significantly associated with the GO term “Synaptic Transmission”. The Variance explained by the first six components in both the validation datasets are provided in Supplementary Figure S2.

##### Figure S2: Variance in gene expression explained by the first six components in the two Validation datasets


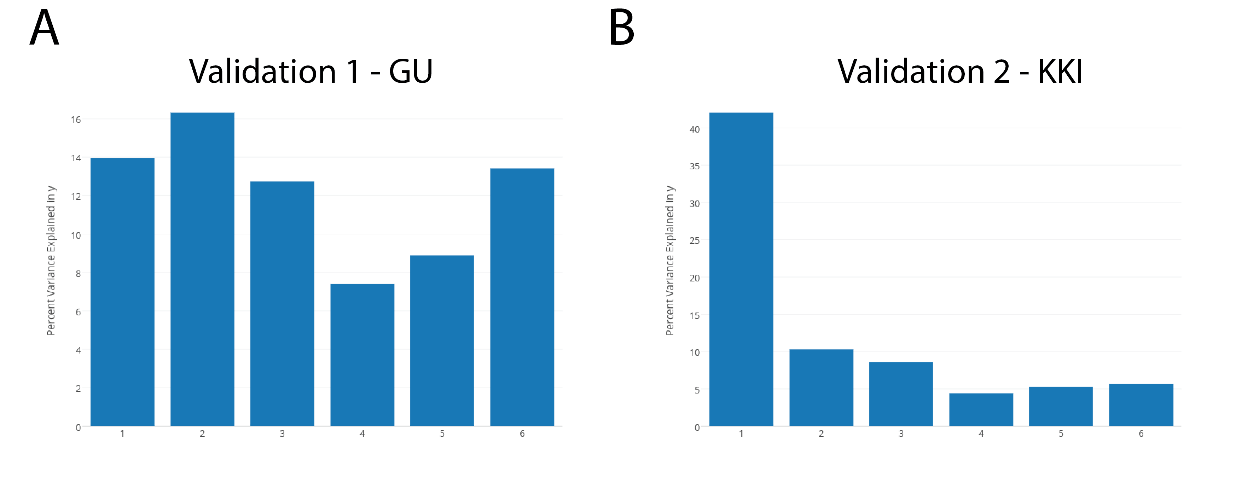


*Only the first component in both the datasets significantly explained variance in ΔCT. Subsequent components all explained less than 10% of the variance in gene expression.*

In comparison with the Discovery dataset, both the validation datasets also had a high, significant correlations in the gene loadings (Supplementary Figure S3)

##### Figure S3: Correlations between gene loadings in all three datasets


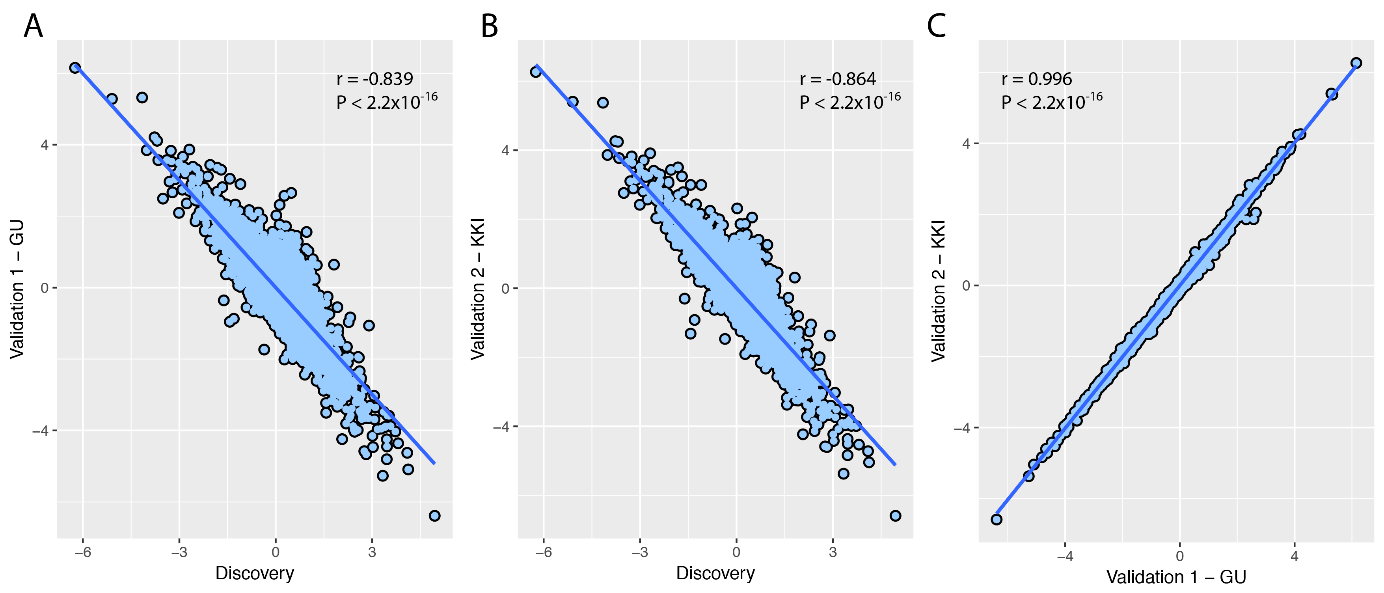


*Correlations between the gene loadings provided for all three datasets. Panel A provides the correlations between the Discovery and Validation 1 datasets. Panel B provides the correlations between the Discovery and Validation 2 datasets. Panel C provides the correlation between the two validation datasets. Only the correlations in ΔCT between the two validation datasets was significant and positive, explaining the negative correlation in gene loadings between the Discovery and two validation datasets.*

We also conducted KEGG based pathway enrichment for the two validation datasets using Enrichr. Details are provided in Supplementary Tables S3A and S3B.

##### Table S3A: Top 10 pathways (Validation 1)

| Term | Overlap | P-value | Adjusted P-value | Z-score |
| --- | --- | --- | --- | --- |
| Adrenergic signaling in cardiomyocytes | 72/148 | 1.142E-04 | 0.0331 | -1.8592 |
| Oxytocin signaling pathway | 73/158 | 7.157E-04 | 0.0899 | -1.9448 |
| Long-term potentiationsapiens_hsa04720 | 35/66 | 9.300E-04 | 0.0899 | -1.8231 |
| MAPK signaling pathway | 109/255 | 1.521E-03 | 0.1103 | -1.9214 |
| Glioma_Homo sapiens | 33/65 | 3.279E-03 | 0.1310 | -1.9054 |
| Retrograde endocannabinoid signaling | 48/101 | 2.699E-03 | 0.1310 | -1.8263 |
| mTOR signaling pathway | 31/60 | 3.048E-03 | 0.1310 | -1.7946 |
| cAMP signaling pathway | 85/199 | 4.792E-03 | 0.1310 | -1.7119 |
| Taste transduction | 40/83 | 4.315E-03 | 0.1310 | -1.6758 |
| Morphine addiction | 43/91 | 4.970E-03 | 0.1310 | -1.5714 |

##### Table S3B: Top 10 pathways (Validation 2)

| **Term** | **Overlap** | **P-value** | **Adjusted P-value** | **Z-score** |
| --- | --- | --- | --- | --- |
| Retrograde endocannabinoid signaling | 78/101 | 1.34E-05 | 0.00 | -1.94 |
| Rap1 signaling pathway | 149/211 | 2.15E-05 | 0.00 | -1.94 |
| Oxytocin signaling pathway | 114/158 | 4.36E-05 | 0.00 | -1.92 |
| Glutamatergic synapse | 84/114 | 1.31E-04 | 0.01 | -1.86 |
| Circadian entrainment | 71/95 | 2.06E-04 | 0.01 | -1.80 |
| Long-term potentiation | 52/66 | 1.47E-04 | 0.01 | -1.75 |
| Thyroid hormone signaling pathway | 86/118 | 2.07E-04 | 0.01 | -1.70 |
| Glioma | 50/65 | 5.60E-04 | 0.01 | -1.75 |
| cAMP signaling pathway | 136/199 | 5.03E-04 | 0.01 | -1.74 |
| Adrenergic signaling in cardiomyocytes | 104/148 | 4.77E-04 | 0.01 | -1.68 |

*Tables 3A and 3B provide the top 10 pathways for the Validation analyses. We find an enrichment for similar neural pathways between the three datasets (Oxytocin signalling pathway, retrograde endocannabinoid signalling, morphine addiction, circadian entertainment, and different neurotransmitter signalling pathways).*

We also investigated if the including only males in either of the two Validation cohort will improve the correlations in ΔCT, gene scores, and gene loadings between the Discovery and the Validation. We conducted this analysis using Validation 2 (KKI) as it had more number of male participants than the Validation 1 dataset. We note that the correlations in ΔCT, gene scores, and gene loadings between the Discovery and the males-only ΔCT were similar to the correlations when males and females were included in Validation 2 (Supplementary Figure S4). Hence, the inclusion of females does not seem to alter the results.

##### Figure S4: Correlations between ΔCT, gene scores and gene loadings between the Discovery and the males-only Validation 2


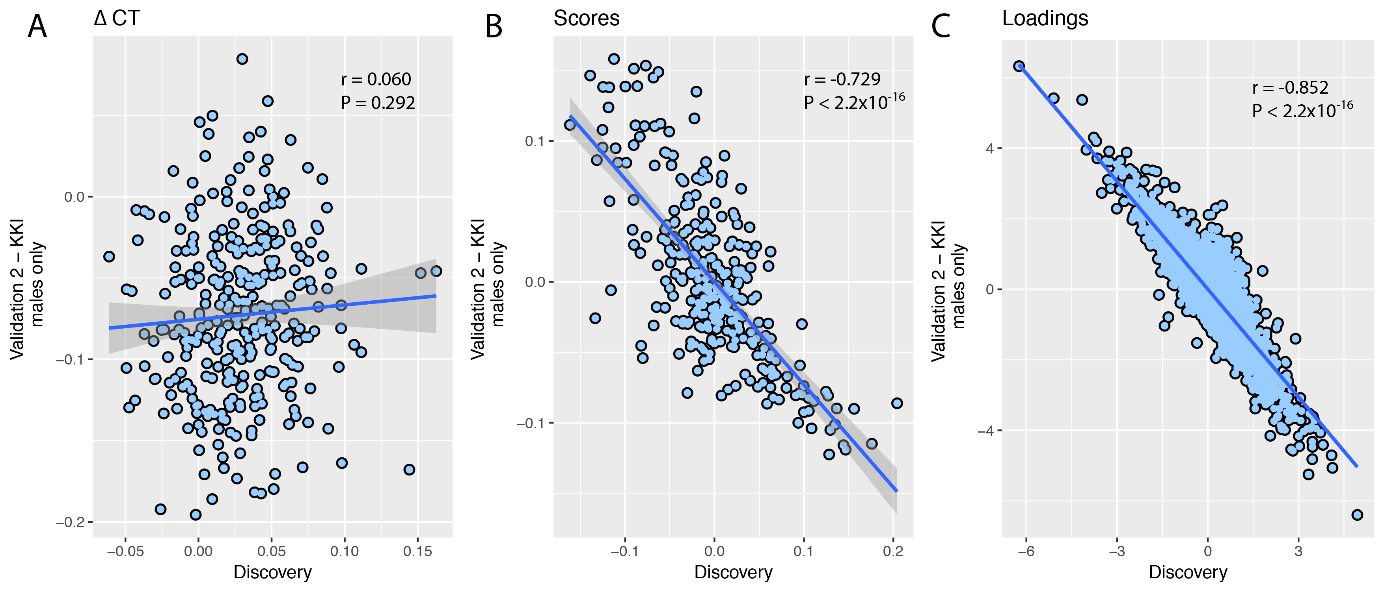


## 5. Von Economo profiling

The spatial profile of the transcriptionally downregulated genes in the autism were examined using a regional map based on the cytoarchitectonic criterion of Von Economo (Von Economo and Koskinas, 2008). In his seminal work, Von Economo described five fundamental types of cortical structures: granular primary motor cortex (class 1), frontal granular association cortex (class 2), parietal homotypical association cortex (class 3), dysgranular secondary sensory cortex (class 4), agranular primary sensory cortex (class 5). Allocortex (class 6) and insular cortex (class 7) were added due to their particular cytoarchitectonical characteristics (Whitaker et al., 2016; Zilles and Amunts, 2012). The average expression of all significant genes in the first PLSR component was calculated for each class (FDR adjusted P-values < 0.05). A non-parametric permutation test was applied to assess whether gene expression values were different from 0 in each class. A reference distribution was created by computing the gene expression values across Von Economo classes for a random subset of genes (10,000 permutations). The two tails of the resulting distribution were used to retain or reject the null hypothesis of an average gene expression equal to 0. Von Economo profiling analysis for the two validation datasets are provided in Supplementary Figure S4.

##### Figure S5: Von Economo Expression Z-Scores of the two validation datasets


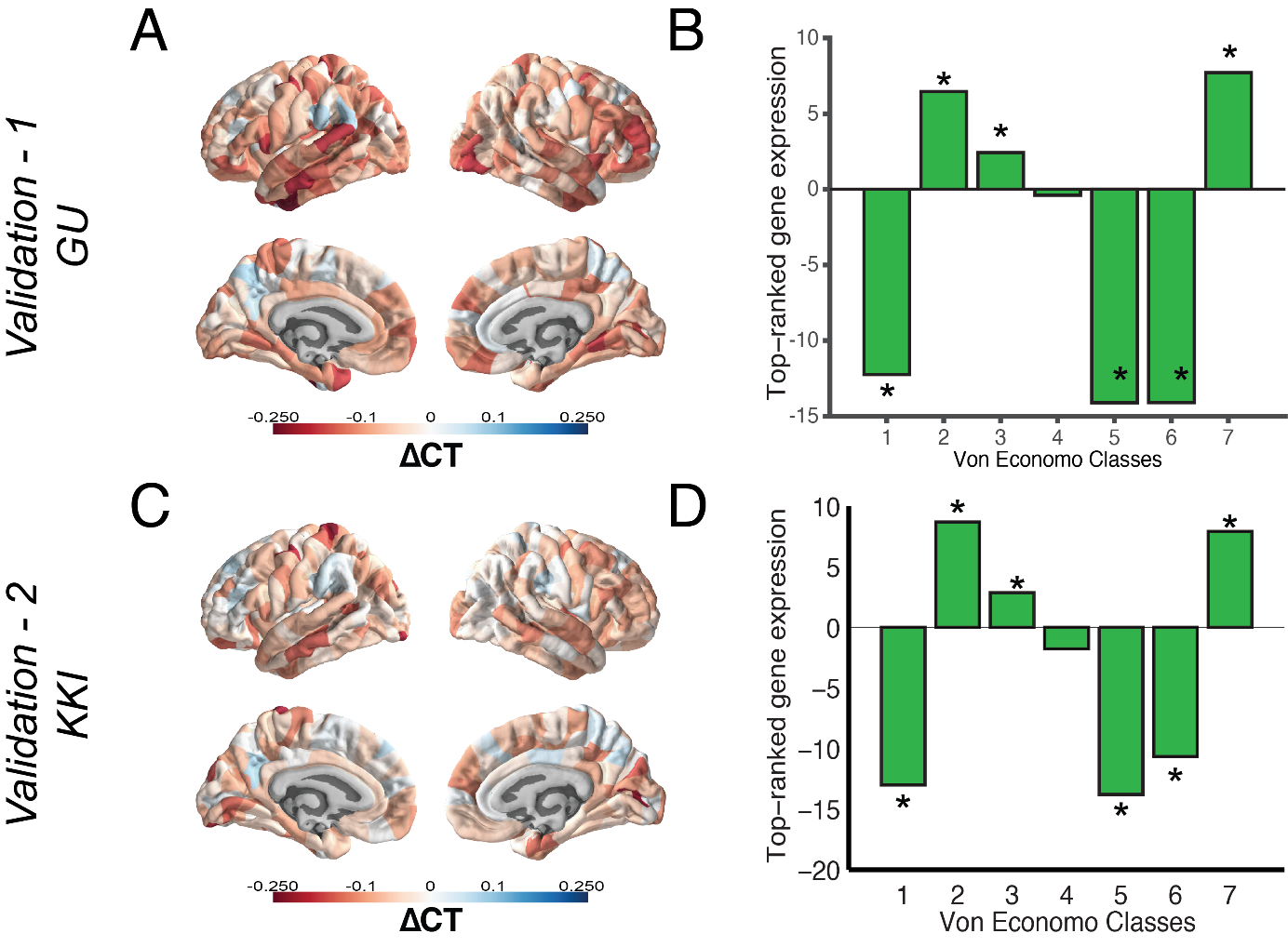


*Von Economo expression profiles largely resemble the pattern of over-expression in association cortices observed in the discovery dataset. The validation datasets reveals a significant over-expression of the PLSR1 in classes 2 and 3 (association cortices), as well as in the insular cortex (class 7). On the other hand, Class 1 and 5 (primary cortices) and Class 6 (limbic regions) show a significant PLSR1 under-expression.*

## 6. PLSR Analysis: ADHD data

To investigate if our results were autism specific we also performed a PLSR analysis on the cortical thickness difference between a matched group of children with ADHD (matched on age, IQ and scanner site across all three groups) and the same neurotypical control group used in the main study. We used the exact same pipeline as described in the main manuscript. Cross-validation results of the initial 35-component PLSR model are listed in Supplementary Table S4. It is immediately clear that this model does not provide a good fit for the ADHD ΔCT as the cross-validated cumulative Q2 does not show a clear peak. Although there are three components that explain more than 10% of the variance each none of these components are significantly associated with the ΔCT. For a full comparison, we nonetheless performed pathway and ontology analyses for those three components revealed no significant pathways or biological processes associated with genes that passed FDR correction in each PLSR component. We did not proceed with further enrichment analyses.

##### Table S5: PLS 35 component model for the ADHD-controls CT difference

| COMP | PRESS | RSS | Q2 | Q2cum |
| --- | --- | --- | --- | --- |
| 1 | 3.14E+02 | 3.07E+02 | -0.0239 | -0.0239 |
| 2 | 2.98E+02 | 2.84E+02 | -0.0481 | -0.0732 |
| 3 | 2.66E+02 | 2.57E+02 | -0.0384 | -0.1144 |
| 4 | 1.89E+02 | 2.04E+02 | 0.0744 | -0.0314 |
| 5 | 1.56E+02 | 1.65E+02 | 0.0570 | 0.0273 |
| 6 | 1.36E+02 | 1.43E+02 | 0.0529 | 0.0787 |
| 7 | 1.26E+02 | 1.18E+02 | -0.0634 | 0.0203 |
| 8 | 8.28E+01 | 7.89E+01 | -0.0492 | -0.0278 |
| 9 | 6.62E+01 | 6.87E+01 | 0.0356 | 0.0087 |
| 10 | 6.54E+01 | 6.11E+01 | -0.0700 | -0.0606 |
| 11 | 5.03E+01 | 5.36E+01 | 0.0603 | 0.0033 |
| 12 | 4.29E+01 | 4.35E+01 | 0.0120 | 0.0153 |
| 13 | 4.01E+01 | 3.84E+01 | -0.0423 | -0.0264 |
| 14 | 3.02E+01 | 2.87E+01 | -0.0548 | -0.0826 |
| 15 | 2.51E+01 | 2.39E+01 | -0.0517 | -0.1385 |
| 16 | 2.06E+01 | 1.88E+01 | -0.0949 | -0.2466 |
| 17 | 1.58E+01 | 1.51E+01 | -0.0463 | -0.3044 |
| 18 | 1.30E+01 | 1.23E+01 | -0.0620 | -0.3852 |
| 19 | 1.05E+01 | 9.71E+00 | -0.0819 | -0.4986 |
| 20 | 7.50E+00 | 6.74E+00 | -0.1126 | -0.6673 |
| 21 | 5.64E+00 | 5.49E+00 | -0.0268 | -0.7120 |
| 22 | 4.64E+00 | 3.96E+00 | -0.1713 | -1.0052 |
| 23 | 3.28E+00 | 3.06E+00 | -0.0720 | -1.1496 |
| 24 | 2.51E+00 | 2.01E+00 | -0.2496 | -1.6861 |
| 25 | 1.63E+00 | 1.31E+00 | -0.2463 | -2.3476 |
| 26 | 1.04E+00 | 9.13E-01 | -0.1341 | -2.7965 |
| 27 | 7.33E-01 | 6.50E-01 | -0.1273 | -3.2798 |
| 28 | 5.68E-01 | 4.95E-01 | -0.1480 | -3.9132 |
| 29 | 4.07E-01 | 3.23E-01 | -0.2594 | -5.1879 |
| 30 | 2.67E-01 | 2.36E-01 | -0.1313 | -6.0006 |
| 31 | 2.01E-01 | 1.53E-01 | -0.3138 | -8.1973 |
| 32 | 1.16E-01 | 9.63E-02 | -0.2034 | -10.0677 |
| 33 | 5.71E-02 | 5.02E-02 | -0.1358 | -11.5707 |
| 34 | 3.41E-02 | 2.96E-02 | -0.1514 | -13.4742 |
| 35 | 1.85E-02 | 1.67E-02 | -0.1073 | -15.0269 |

*For each component the Predictive Error Sum of Squares (PRESS), Residual Sum of Squares (RSS), cross validated PRESS (Q2) and the cumulative Q2 are provided.*

## 7. Gene modules and enrichment analyses

#### Transcriptional dataset and adult gene co-expression modules

*The following describes the analyses conducted in Parikshak et al., 2016. We also outline our rationale for using these datasets in the present study.*

Briefly, rRNA-depleted RNA sequencing was conducted using cortical brain tissue samples from 48 autism donors and 49 neurotypical controls. Differential gene expression (DGE) identified 1143 dysregulated genes in the autism cortex samples compared to the control cortex samples of which 584 genes were upregulated and 558 genes were downregulated in the autism cortex. This dataset comprises of 13 autism donors and 14 control donors that overlap with Gandal et al., 2016 due to the inclusion of data from Voineagu et al., 2011. DGE analyses were performed using gene expression levels that have been normalized for gene length, library size, and G+C content. DGE was calculated using a linear mixed effects regression model where individual donor identifier was treated as a random effect, and age, sex, brain region, and diagnosis were treated as fixed effects. Genes were said to be differentially expressed if they had a Benjamini-Hochberg FDR corrected P < 0.05. In our analyses, we used genes and associated P-values and fold difference from Supplementary Table 2 from Parikshak et al., 2016 using the cortex-only dataset. We defined genes as being transcriptionally dysregulated if they had a Benjamini-Hochberg FDR corrected P-value < 0.05. In this subset of significant genes, genes were downregulated if they had a log2(Fold-change) < 0, and similarly, they were upregulated if they had a log2(Fold-change) > 0. Downregulated genes were significantly enriched for pathways involved in synaptic transmission and genes expression in neurons.

Weighted gene co-expression modules were constructed using the R package weighted gene co-expression network analyses (WGCNA) (https://labs.genetics.ucla.edu/horvath/CoexpressionNetwork/Rpackages/WGCNA/) after bootstrapping 100 times. Modules significantly associated with a diagnosis of autism were identified using a linear mixed effects regression analyses, using the first principal component of each module against diagnosis, age, sex, and brain region. WGCNA is an excellent data-reduction technique, that utilizes gene co-expression patterns to construct weighted co-expression network modules. This identifies clusters of genes with similar expression that are usually enriched for specific biological pathways. Here we focussed on six gene co-expression modules constructed from the entire cortical gene-expression profile that are associated with autism: M9, M19, and M20 that are enriched for upregulated genes in autism, and M4, M10, and M16 that are enriched for downregulated genes in autism. All six modules show significant cell-type enrichment. Further, the downregulated modules are all enriched for synaptic function and neuronal genes and the upregulated modules are enriched for inflammatory pathway and glial function. As we had identified a significant enrichment of synaptic function in the significant PLSR1 genes we hypothesized that genes would be enriched for the three downregulated modules. This further supported the enrichment of the PLSR1 genes for the transcriptionally dysregulated genes in the autism post-mortem cortex.

#### Validation Transcriptional dataset

*The following describes the analyses conducted in Gandal et al., 2016.*

DGE analysis was conducted using raw microarray gene expression data from cortical samples from 33 autism donors and 38 control donors. Three autism microarray datasets were used from three different studies: (Chow et al., 2012; Garbett et al., 2008; Voineagu et al., 2011). Samples from the Voineagu et al., 2011 overlapped with the Parikshak et al., 2016 dataset, making this a quasi-independent dataset. DGE was calculated using the log fold change values using the Limma package after accounting for biological covariates (age, sex, brain region) and technical covariates (such as experimental batch, post-mortem interval, pH etc) for each study separately. Finally, meta-analysis of the log fold change values were conducted using a random effects model. Approximately 10,000 genes were retained after filtering for genes that were present across all three studies. As mentioned above, we identified genes as being downregulated if they had a log(Fold-change) < 0, and an Benjamini-Hochberg FDR corrected P-value < 0.05. We identified 830 transcriptionally dysregulated genes, and 464 genes that were downregulated in the autism postmortem cortex.

#### Developmental gene co-expression modules

*The following describes the analyses conducted in Parikshak et al., 2013.*

WGCNA network analyses were conducted using RNA sequencing based gene expression data from BrainSpan whole-genome transcriptomic data. The authors used only data from brain samples spanning 8 weeks post-conception to 12 months after birth. WGCNA identified a total of 17co-expresion modules. Five of these modules are associated with different risks for autism. Modules Mdev 13, Mdev16, and Mdev17 are enriched for transcriptionally dysregulated genes in the autism post-mortem cortex. Whereas modules Mdev2 and Mdev3 are enriched for rare genetic variants associated with autism. Modules Mdev13, Mdev16, and Mdev17 are enriched for the GO term ‘Synaptic transmission’ and are all upregulated. Mdev16 is upregulated first (Post-conception week - PCW 10), followed by Mdev17 (PCW 13) and finally Mdev13 (PCW 16). The developmental trajectories of all three modules are closely aligned but are sequential. In contrast, Mdev2 and Mdev3 are enriched for pathways associated with DNA binding and transcriptional regulation.

#### Rare de novo genetic variants

*The following describes the analyses conducted in* Sanders et al., 2015*.*

Genes harbouring rare, de novo variants associated with autism were identified from Sanders et al., 2015. A total of 65 genes associated with autism (FDR adjusted P-value < 0.1) were identified using exome sequencing data and small de novo CNV detection largely using genotyping data. This was conducted after integrating data from multiple different studies from more than 10,220 individuals from 2,591 families recruited as a part of the Simon’s Simplex Collection.

#### Common genetic variants

Genome-wide association data of the latest data freeze of the autism spectrum disorder working group of the Psychiatric Genomics Consortium was downloaded from the PGC website (<http://www.med.unc.edu/pgc/results-and-downloads>). This dataset consists of 5,305 cases and 5,305 pseudocontrols (i.e. the non-transmitted haplotypes of the parents) of European ancestry. A total of 553,795,981 SNPs were tested for association. SNP based P-values were converted to gene-based P-values using a hg19 genome build using MAGMA (de Leeuw et al., 2015), which accounts for linkage disequilibrium between SNPs when calculating gene-based P-values. The Benjamini-Hochberg FDR corrected gene-based P-values were used as the independent variable to test for enrichment analyses using a regression model (explained below).

### Regression analyses

Usually, enrichment analyses are conducted using Fisher’s exact test or hypergeometric test (equivalent to a one-sided Fisher’s exact test). However, these tests do not incorporate potential covariates that may arise with the different methods used to identify significant genes (for example, comparing enrichment of genes identified using exome sequencing in a list of genes identified using RNA expression). One significant covariate for enrichment analyses is gene length. To correct for the gene length bias we used a logistic regression analyses with gene length included as a covariate.This can be written as:

Y = B_0_ + B_1_x_1_ + B_2_x_2_ + E

Where Y is the odds that the gene is significant after FDR correction for the PLSR module (1 if the gene is significant, 0 if the gene is not significant); x1 is the independent variable in the autism dataset tested; x2 is the gene length. Regression analyses were conducted in R. For all enrichment analyses, the dependent variable (Y) was if the gene was significant after FDR correction in the PLSR component. If the gene was significant, i.e. FDR-adjusted P-value < 0.05, this gene was given a membership of 1, and a membership of 0 if the gene failed to reach significant. The independent varied between the analyses. For the transcriptionally regulated gene lists, the independent variable was the absolute fold-change of the gene if the gene was significant after FDR correction (P_corrected < 0.05). For gene co-expression module analyses, the kME for all the genes which measures the module membership of the gene. For the common variants, we used the Z scores of the gene-based P-values. Finally, for the rare-variant analyses, if the gene was one of the 65 genes harbouring rare de novo variants (Sanders et al., 2015), the gene was given a membership of 1, and if not a membership of 0. Results of the enrichment analysis are provided in Supplementary Table S6

##### Table S6: Results of the gene enrichment analyses

| **Discovery (ABIDE 1)** | | | | | | |
| --- | --- | --- | --- | --- | --- | --- |
| **Category** | **Dataset** | **OR** | **Upper CI (95%)** | **Lower CI (95%)** | **P** | **P_corrected** |
| Autism transcription | Dysregulated | 1.21 | 1.23 | 1.19 | 1.76E-15 | 2.81E-15 |
| Autism transcription | Downregulated | 1.87 | 1.94 | 1.8 | 2.00E-16 | 3.55E-16 |
| Autism transcription | Upregulated | 1.01 | 1.02 | 1 | 4.99E-01 | 4.99E-01 |
| Adult co-expression modules | Mod4 | 1.08 | 1.08 | 1.07 | 2.00E-16 | 3.55E-16 |
| Adult co-expression modules | Mod10 | 1.07 | 1.08 | 1.07 | 2.00E-16 | 3.55E-16 |
| Adult co-expression modules | Mod16 | 1.08 | 1.08 | 1.07 | 2.00E-16 | 3.55E-16 |
| Adult co-expression modules | Mod9 | 0.93 | 0.94 | 0.92 | 2.01E-14 | 2.92E-14 |
| Adult co-expression modules | Mod19 | 0.93 | 0.94 | 0.92 | 2.00E-16 | 3.55E-16 |
| Adult co-expression modules | Mod20 | 0.97 | 0.97 | 0.96 | 6.22E-05 | 7.66E-05 |
| Common variants | Common variants | 1 | 1.01 | 1 | 2.75E-01 | 2.93E-01 |
| Rare variants | Rare variants | 0.96 | 0.99 | 0.93 | 2.42E-01 | 2.76E-01 |
| Fetal co-expression modules | Moddev2 | 0.97 | 0.97 | 0.96 | 1.28E-11 | 1.70E-11 |
| Fetal co-expression modules | Moddev3 | 0.96 | 0.97 | 0.96 | 2.00E-16 | 3.55E-16 |
| Fetal co-expression modules | Moddev13 | 1.04 | 1.04 | 1.04 | 2.00E-16 | 3.55E-16 |
| Fetal co-expression modules | Moddev16 | 1.06 | 1.06 | 1.05 | 2.00E-16 | 3.55E-16 |
| Fetal co-expression modules | Moddev17 | 1.04 | 1.05 | 1.04 | 2.00E-16 | 3.55E-16 |
| **Validation 1 (GU)** | | | | | | |
| **Category** | **Dataset** | **OR** | **Upper CI (95%)** | **Lower CI (95%)** | **P** | **P_corrected** |
| Autism transcription | Downregulated | 1.24 | 1.31 | 1.18 | 4.05E-03 | 4.62E-03 |
| Adult co-expression modules | Mod4 | 1.06 | 1.07 | 1.04 | 2.49E-05 | 3.32E-05 |
| Adult co-expression modules | Mod10 | 1.05 | 1.06 | 1.04 | 2.02E-05 | 3.23E-05 |
| Adult co-expression modules | Mod16 | 1.07 | 1.08 | 1.06 | 2.81E-10 | 5.62E-10 |
| Fetal co-expression modules | Moddev13 | 1.05 | 1.06 | 1.05 | 8.85E-11 | 2.36E-10 |
| Fetal co-expression modules | Moddev16 | 1.07 | 1.08 | 1.06 | 3.47E-12 | 1.39E-11 |
| Fetal co-expression modules | Moddev17 | 1.07 | 1.07 | 1.06 | 2.25E-14 | 1.80E-13 |
| **Validation 2 (KKI)** | | | | | | |
| **Category** | **Dataset** | **OR** | **Upper CI (95%)** | **Lower CI (95%)** | **P** | **P_corrected** |
| Autism transcription | Downregulated | 1.3 | 1.45 | 1.15 | 6.35E-04 | 1.10E-03 |
| Adult co-expression modules | Mod4 | 1.02 | 1.04 | 0.99 | 1.95E-01 | 1.95E-01 |
| Adult co-expression modules | Mod10 | 1.04 | 1.06 | 1.01 | 2.48E-03 | 3.00E-03 |
| Adult co-expression modules | Mod16 | 1.02 | 1.04 | 1 | 3.16E-02 | 3.60E-02 |
| Fetal co-expression modules | Moddev13 | 1.06 | 1.07 | 1.04 | 6.20E-11 | 4.34E-10 |
| Fetal co-expression modules | Moddev16 | 1.05 | 1.05 | 1.05 | 1.69E-06 | 3.94E-06 |
| Fetal co-expression modules | Moddev17 | 1.05 | 1.07 | 1.04 | 3.04E-09 | 1.06E-08 |
| **Validation_transcription** | | | | | | |
| **Dataset1** | **Dataset2** | **OR** | **Upper CI (95%)** | **Lower CI (95%)** | **P** | **P_corrected** |
| Discovery MRI | Downregulated genes | 1.4 | 1.45 | 1.34 | 6.19E-08 | 1.86E-07 |
| Validation1 MRI | Downregulated genes | 1.35 | 1.44 | 1.27 | 3.68E-03 | 5.00E-03 |
| Validation2 MRI | Downregulated genes | 1.3 | 1.52 | 1.08 | 1.97E-02 | 1.90E-02 |

## 8. Third validation dataset

Although no comparable dataset exists the NYU scanner site provides a relatively large dataset (29 neurotypical subjects, 75 individuals with autism) with a slightly larger age range but comparable mean (age control 9.57±3.37, age autism 8.88±4.88). Although this data is not the ideal comparison to our original three datasets due to the explicit inclusion of older individuals we nonetheless re-ran our PLS analysis on this dataset and confirmed the delta CT correlation to the two initial validation datasets. We also compared the PLS scores obtained from this 4^th^ dataset to both our PLS datasets and confirmed again the correlation between delta CT and PLS scores as per our original analysis. Results are summarized in figure S6 below.

##### Figure S6: third validation dataset


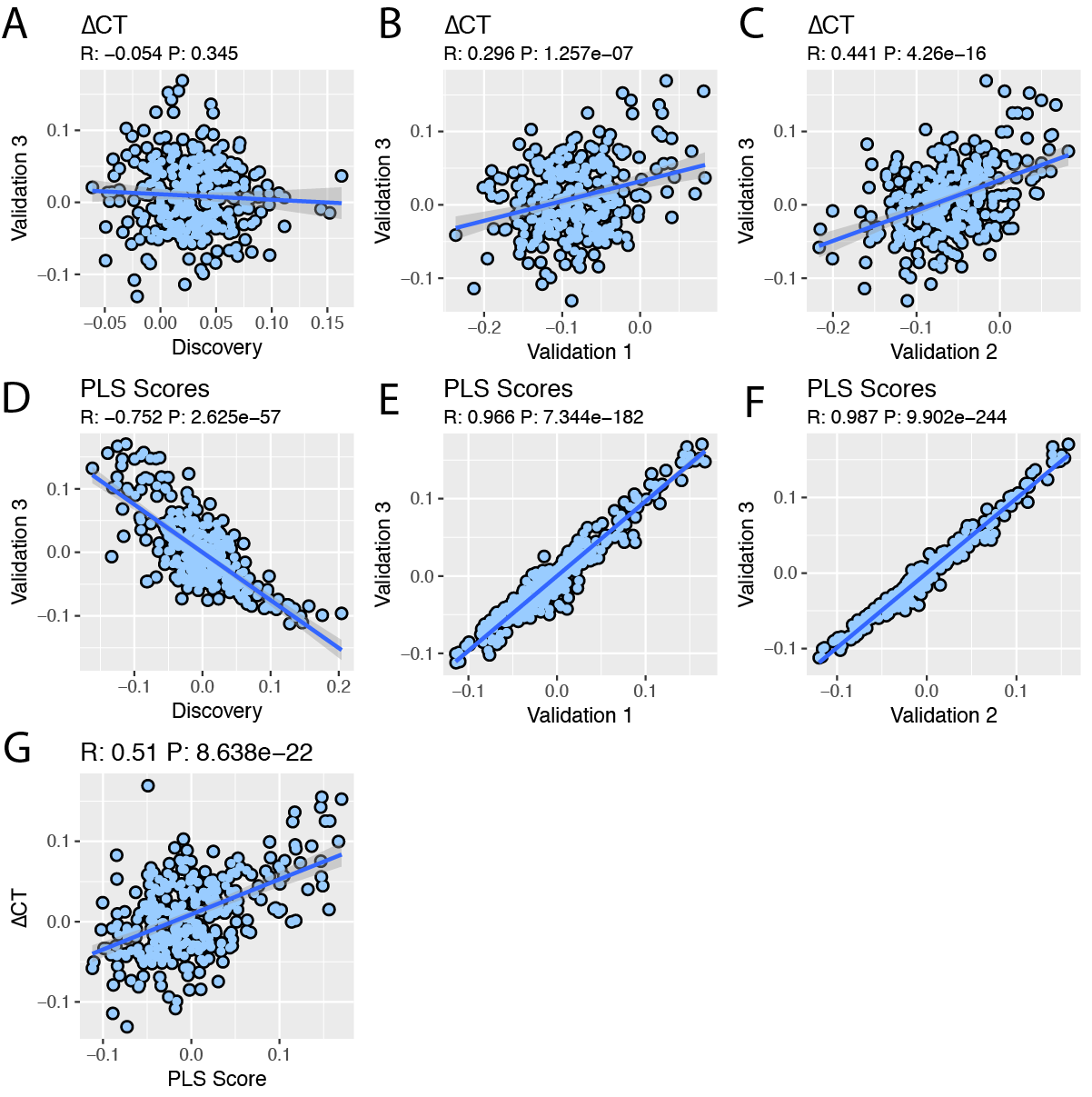


Panels A-C show the correlation between ∆CT for the third validation dataset against the other three datasets. Indicating consistent correlations as reported in Figure 3. Panels D-F show the correlation between the PLSR scores of all three datasets, again consistent with Figure 3. Panels G show sthe correlation between ∆CT and the PLSR scores for the third validation dataset again being consistent with panels G-I in Figure 3 of the main manuscript.

## References

Bethlehem, R.A.I., Romero-Garcia, R., Mak, F.K., Bullmore, E.T., and Baron-Cohen, S. (2017). Structural covariance networks in children with autism or ADHD. *Cerebr. Cortex*.

Chen, C., Grennan, K., Badner, J., Zhang, D., Gershon, E., Jin, L., Liu, C., 2011. Removing batch effects in analysis of expression microarray data: An evaluation of six batch adjustment methods. PLoS One 6. doi:10.1371/journal.pone.0017238

Chow, M.L., Pramparo, T., Winn, M.E., Barnes, C.C., Li, H.R., Weiss, L., Fan, J.B., Murray, S., April, C., Belinson, H., et al. (2012). Age-dependent brain gene expression and copy number anomalies in autism suggest distinct pathological processes at young versus mature ages. PLoS Genet. *8*.

Desikan, R.S., Ségonne, F., Fischl, B., Quinn, B.T., Dickerson, B.C., Blacker, D., Buckner, R.L., Dale, A.M., Maguire, R.P., Hyman, B.T., et al. (2006). An automated labeling system for subdividing the human cerebral cortex on MRI scans into gyral based regions of interest. Neuroimage *31*, 968–980.

Von Economo, C., and Koskinas, G.N. (2008). Atlas of Cytoarchitectonics of the Adult Human Cerebral Cortex (Thessaloniki: Karger).

Gandal, M.J., Haney, J., Parikshak, N., Leppa, V., Horvath, S., and Geschwind, D.H. (2016). Shared molecular neuropathology across major psychiatric disorders parallels polygenic overlap. bioRxiv.

Garbett, K., Ebert, P.J., Mitchell, A., Lintas, C., Manzi, B., Mirnics, K., and Persico, A.M. (2008). Immune transcriptome alterations in the temporal cortex of subjects with autism. Neurobiol. Dis. *30*, 303–311.

Johnson, W.E., Li, C., Rabinovic, A., 2007. Adjusting batch effects in microarray expression data using empirical Bayes methods. Biostatistics 8, 118–127. doi:10.1093/biostatistics/kxj037

de Jong, S. (1993). SIMPLS: An alternative approach to partial least squares regression. Chemom. Intell. Lab. Syst. *18*, 251–263.

de Leeuw, C.A., Mooij, J.M., Heskes, T., and Posthuma, D. (2015). MAGMA: Generalized Gene-Set Analysis of GWAS Data. PLoS Comput. Biol. *11*, 1–19.

Parikshak, N.N., Luo, R., Zhang, A., Won, H., Lowe, J.K., Chandran, V., Horvath, S., and Geschwind, D.H. (2013). Integrative Functional Genomic Analyses Implicate Specific Molecular Pathways and Circuits in Autism. Cell *155*, 1008–1021.

Parikshak, N.N., Swarup, V., Belgard, T.G., Irimia, M., Ramaswami, G., Gandal, M.J., Hartl, C., Leppa, V., Ubieta, L.T., Huang, J., et al. (2016). Genome-wide changes in lncRNA, splicing, and regional gene expression patterns in autism. Nature *540*, 423–427.

Romero-garcia, R., Atienza, M., Clemmensen, L.H., and Cantero, J.L. (2012). Effects of network resolution on topological properties of human neocortex. Neuroimage *59*, 3522–3532.

Sanders, S.J., He, X., Willsey, A.J., Ercan-Sencicek, A.G., Samocha, K.E., Cicek, A.E., Murtha, M.T., Bal, V.H., Bishop, S.L., Dong, S., et al. (2015). Insights into Autism Spectrum Disorder Genomic Architecture and Biology from 71 Risk Loci. Neuron *87*, 1215–1233.

Voineagu, I., Wang, X., Johnston, P., Lowe, J.K., Tian, Y., Horvath, S., Mill, J., Cantor, R.M., Blencowe, B.J., and Geschwind, D.H. (2011). Transcriptomic analysis of autistic brain reveals convergent molecular pathology. Nature *474*, 380–384.

Whitaker, K.J., Vértes, P.E., Romero-Garcia, R., Váša, F., Moutoussis, M., Prabhu, G., Weiskopf, N., Callaghan, M.F., Wagstyl, K., Rittman, T., et al. (2016). Adolescence is associated with transcriptionally patterned consolidation of the hubs of the human brain connectome. Proc. Natl. Acad. Sci. 2–7.

Wold, S., Sjöström, M., and Eriksson, L. (2001). PLS-regression: A basic tool of chemometrics. Chemom. Intell. Lab. Syst. *58*, 109–130.

Zilles, K., and Amunts, K. (2012). Segregation and Wiring in the Brain. Science (80-. ). *335*, 1582–1584.
